# Unexpected insertion of carrier DNA sequences into the fission yeast genome during CRISPR–Cas9 mediated gene deletion

**DOI:** 10.1186/s13104-019-4228-x

**Published:** 2019-03-29

**Authors:** Sophie Longmuir, Nabihah Akhtar, Stuart A. MacNeill

**Affiliations:** School of Biology, University of St Andrews, North Haugh, St Andrews, Fife, KY16 9ST UK

**Keywords:** Microprotein, Fission yeast, *Schizosaccharomyces pombe*, *Oncorhynchus keta*, CRISPR–Cas9

## Abstract

**Objectives:**

The fission yeast *Schizosaccharomyces pombe* is predicted to encode ~ 200 proteins of < 100 amino acids, including a number of previously uncharacterised proteins that are found conserved in related *Schizosaccharomyces* species only. To begin an investigation of the function of four of these so-called microproteins (designated Smp1–Smp4), CRISPR–Cas9 genome editing technology was used to delete the corresponding genes in haploid fission yeast cells.

**Results:**

None of the four microprotein-encoding genes was essential for viability, meiosis or sporulation, and the deletion cells were no more sensitive to a range of cell stressors than wild-type, leaving the function of the proteins unresolved. During CRISPR–Cas9 editing however, a number of strains were isolated in which additional sequences were inserted into the target loci at the Cas9 cut site. Sequencing of the inserts revealed these to be derived from the chum salmon *Oncorhynchus keta*, the source of the carrier DNA used in the *S. pombe* transformation.

**Electronic supplementary material:**

The online version of this article (10.1186/s13104-019-4228-x) contains supplementary material, which is available to authorized users.

## Introduction

Microproteins (also known as SEPs for smORF-encoded peptides) are small (generally < 100 amino acid) proteins that are increasingly being implicated in a wide range of biological processes in all three domains of life [1]. The genome of the unicellular fission yeast *Schizosaccharomyces pombe* potentially encodes > 200 proteins of less than 100 amino acids in length, of which 36 are annotated in the PomBase database as being essential and 100 as non-essential [2]. These include well-characterised proteins functioning in DNA replication, transcription, translation (including > 20 ribosomal subunits), RNA splicing and processing, electron transport, ATP synthesis, cell mating and protein modification [2]. The status of the remaining ~ 100 microprotein-encoding smORFs is unknown and it remains possible that some are not actually protein coding.

The results presented here arose out of a project to investigate the function of four unstudied *S. pombe* microproteins, designated Smp1–Smp4 (see Table 1 for systematic IDs). Each of these proteins is conserved to a greater or lesser extent in the three other *Schizosaccharomyces* species whose genomes have been sequenced [3], each is unique to the *Schizosaccharomyces*, and each has been detected in quantitative proteomic studies as being present at between 1800 and 10,000 molecules per cell in either exponentially growing or G1-arrested haploid cells [4]. To initiate this work, the genes encoding Smp1–Smp4 were deleted from the genome using CRISPR–Cas9 genome editing technology, demonstrating that none of the four proteins is essential for haploid growth. In the course of the CRISPR–Cas9 genome editing however, we identified a number of strains carrying unexpected sequence insertions in the vicinity of the targeted loci. Here, we show that the inserted sequences are derived from the salmon sperm DNA that was used as a carrier during yeast transformation, suggesting that denaturing carrier DNA prior to use in CRISPR–Cas9 editing procedures in yeast remains important even if the boost to transformation efficiency that results from denaturation is not crucial for the subsequent workflow.

**Table 1:** Microproteins under investigation in this study

Protein	Systematic ID	Length	Comments
Smp1	SPAC25B8.20	72 aa	Conserved in Sj. Contains putative coiled-coil region
Smp2	SPBC13G1.16	62 aa	Conserved in Sc.
Smp3	SPBC30B4.09	51 aa	Conserved in Sj, Sc and So. Contains putative transmembrane helix
Smp4	SPCPB16A4.07	69 aa	Conserved in Sj, Sc and So. Contains putative disordered region

Sp, *S. pombe*; So, *S. octosporus*; Sj, *S. japonicus*; Sc, *S. cryophilus*

## Main text

### Methods

#### Fission yeast strains and methods

A full list of fission yeast strains used can be found in Additional file 1: Table S1. Methods for yeast growth and analysis can be found in Additional file 2.

#### Genome editing

For detailed molecular biology methods, see Additional file 2. Primer sequences, plasmids and synthetic DNAs are described in Additional file 1: Tables S2–S4. Briefly, the online tool CRISP4P was used to design primers for ligation-free cloning of sgRNA-coding inserts into the constitutive Cas9-expressing plasmid pJB166. The resulting plasmids were then co-transformed into *fex1 fex2 S. pombe* alongside commercially synthesised homologous recombination templates, with salmon sperm DNA being used as carrier DNA for transformation. After 4 days of growth at 32 °C, the smallest colonies were re-streaked on non-selective medium to allow plasmid loss. Genomic DNA was then prepared from independent single colonies and screened by PCR to identify deletions.

### Results

#### Microproteins in fission yeast

Querying the PomBase database [2] identifies 236 smORFs with the potential to encode microproteins less than 100 amino acids in length. Twenty of these are annotated as being unique to *Schizosaccharomyces* species, with only six of these having been previously studied. In this study we choose to investigate four of the remaining 14 genes, which we designated *smp1*^+^ (*Schizosaccharomyces*-specific microprotein 1, systematic ID SPAC25B8.20), *smp2*^+^ (SPBC13G1.16), *smp3*^+^ (SPBC30B4.09) and *smp4*^+^ (SPCPB16A4.07). The Smp1–Smp4 proteins are 72, 62, 51 and 69 amino acids in length. Transcripts from all four proteins have been detected in both mitotically growing and G1-arrested cells. The Smp1, Smp3 and Smp4 proteins are predicted to contain a short coiled-coil region, a transmembrane helix and a short disordered region, respectively (Table 1). No potential structural features are predicted for the Smp2 protein. None of the proteins is related to any of the others.

#### CRISPR–Cas9 genome editing

To probe the function of the Smp1–Smp4 proteins, we first tested whether it was possible to delete the corresponding genes. To do this, we combined features of three recently described CRISPR–Cas9 methods for *S. pombe* [5–7]. CRISPR4P [6] was used to specify primer sequences for use in ligation-free cloning reactions to generate sgRNA coding inserts for Cas9-encoding plasmid pJB166 [7]. Next, Cas9-sgRNA plasmids were transformed into *S. pombe* cells that had been synchronised in G1 by nitrogen starvation using EMM-N medium, made competent and then cryopreserved [6]. We used a fluoride-sensitive *fex1 fex2 S. pombe* prototroph for these experiments (see Additional file 1: Table S1) to allow for selection of transformants on YE4S supplemented with 1 mM sodium fluoride and to maximise growth rate [7]. Plasmids were co-transformed with commercially synthesised 400 bp gene fragments as homologous recombination (HR) templates. Transformation was achieved using the lithium acetate method, exactly as described [6], with salmon sperm DNA used a carrier. After 4 days of growth at 32 °C, 24–32 of the smallest transformant colonies were individually picked and re-streaked on YE4S to allow loss of the toxic Cas9-encoding pJB166-sgRNA plasmid. Genomic DNA was then prepared from independent single colonies and screened by diagnostic PCR to identify deletions.

**Fig. 1:**
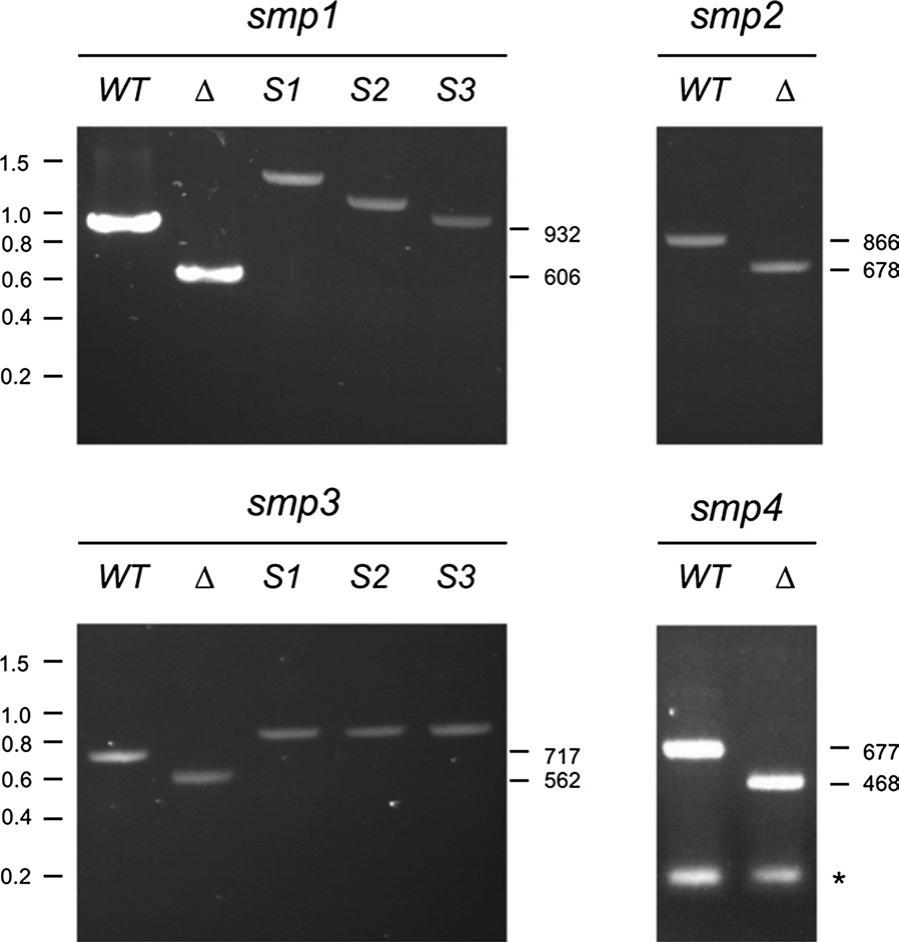
Diagnostic PCR of wild-type (WT), deletion (∆) and insertion (S1–S3) strains for *smp1*–*smp4*. PCR was performed using the primers listed in Additional file 1, Table S2. Molecular weight standard sizes (kb) are shown to the left. Predicted product sizes for wild-type and deletion strains are shown to the right of each gel. The asterisk for *smp4* indicates primer dimers. See text for further details

Three types of colony were identified by PCR (Fig. 1). For each targeted gene, the first type of colony (presenting at least 50% of colonies screened) produced a PCR product that was indistinguishable in size from the wild-type, suggesting that the attempted deletion had not succeeded. (Note that we did not sequence the targeted loci in any of these apparent wild-types, so it remains possible that they contain small insertions or deletions at or around the sgRNA-directed cleavage site). The second class of colony produced a PCR product that was consistent with deletion of the target gene via HR. These colonies represented 3–12% of those screened, consistent with previous observations [6]. In each case, the targeted locus was sequenced to confirm the deletion had occurred as planned, i.e. by homologous recombination involving the supplied HR template. The third type of colony produced either no visible PCR product (despite repeated attempts) or a product of variable size. These constituted a significant number of the colonies analysed: in the case of *smp3* for example, 9 of 24 colonies analysed fell into this category, compared to 12 putative wild-types and three bona fide *smp3∆* deletions.

#### Phenotypic analysis

As it was possible to isolate all four *smp1∆*–*smp4∆* deletion strains, we concluded that all four of the encoded microproteins are non-essential for *S. pombe* haploid growth and division. To examine this in more detail, we measured growth rates and cell length at division for *smp1∆*–*smp4∆* strains during exponential growth on YE4S medium at 32 °C. Growth of all four strains was indistinguishable from the wild-type with a doubling time of ~ 135 min. Similarly, *smp1∆*–*smp4∆* cells underwent cell division at a similar cell size to wild-type (~ 14 µm) and no differences in cell shape were apparent (see Additional file 3: Figure S2). We next explored growth in response to 10 distinct stress conditions (see Additional file 1: Table S5 for details) but once again no significant differences in growth were observed between *smp1∆*–*smp4∆* and the wild-type control. Finally, we tested the ability of *smp1∆*–*smp4∆* strains to undergo mating and sporulation. In each case (*smp1∆* x *smp1∆*, *smp2∆* x *smp2∆*, etc.), efficient mating and production of four-spored zygotic asci was observed (see Additional file 3: Figure S2), indicating that none of these four microproteins is essential for meiosis and sporulation.

#### Characterisation of inserted sequences

**Fig. 2:**
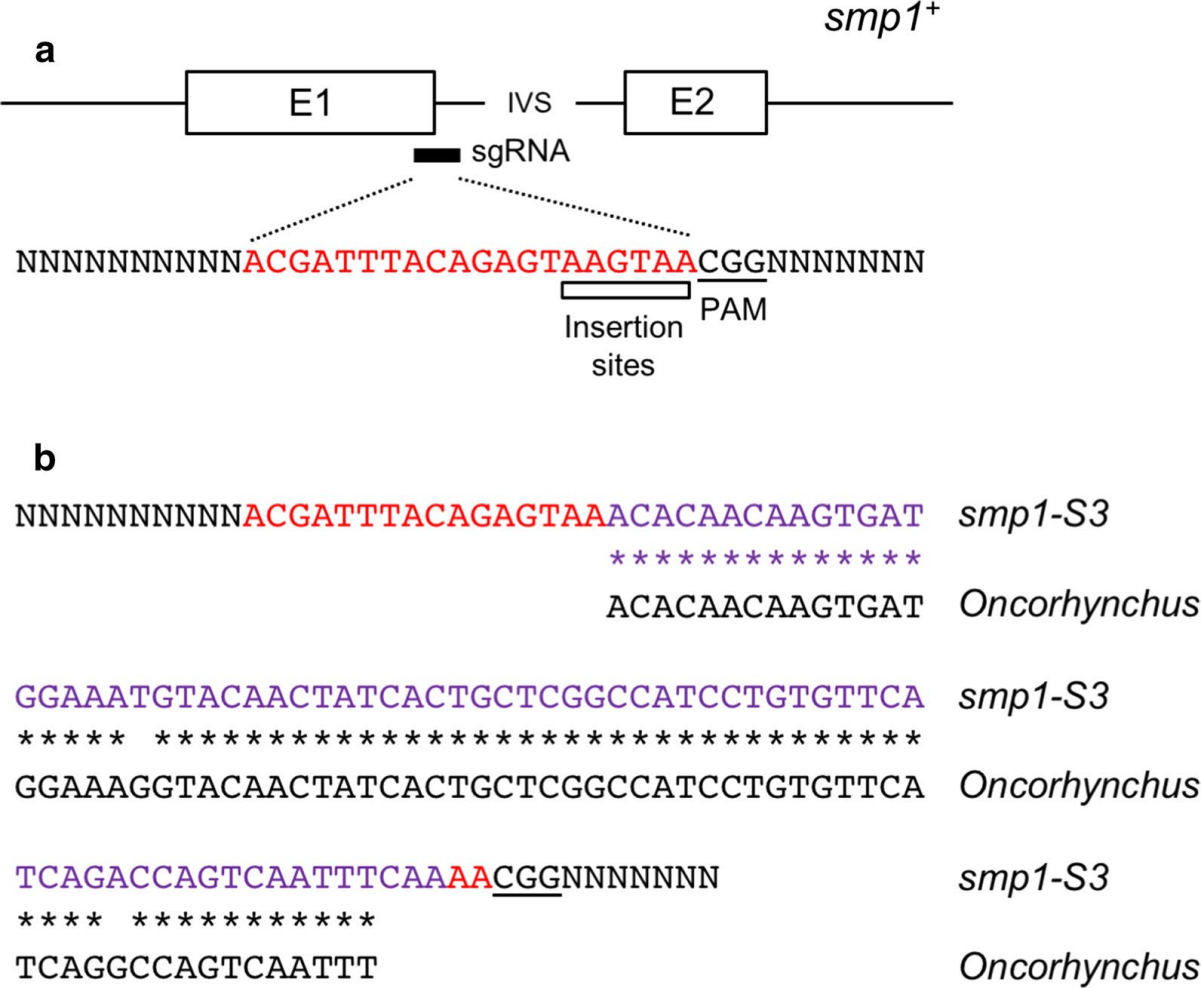
Insertion of *Oncorhynchus* DNA into CRISPR–Cas9 targeted locus. **a** Schematic of *smp1*^+^ gene region showing exons (E1, E2), intervening sequence (IVS), location of sgRNA complementary sequence (shown in red) and PAM site (underlined). **b** Sequence of corresponding region in *smp1*-*S3* insertion allele, showing sgRNA (red), PAM (underlined) and 73 bp inserted sequence closely related (97% identity over 70 nucleotides) to an *Oncorhynchus* genomic sequence, in this case from *O. tshawytscha*, the Chinook salmon (GenBank ID PEKY01000129.1). See also Additional file 1: Table S6 and Additional file 2: Figure S3

As described above, PCR screening to identify *smp1∆*–*smp4∆* strains identified a significant number of strains in which the PCR product obtained was markedly larger than that expected from non-deleted wild-type cells. In order to better understand this phenomenon, reasoning that characterization of these strains might provide insights into mechanisms of off-target effects of CRISPR–Cas9 in *S. pombe*, six strains (three *smp1* and three *smp3* strains) were analysed further by amplifying genomic DNA, sequencing the resulting PCR product and searching sequence databases to identify the source of the inserted DNA. In each case that we examined, the additional sequences (which varied from 73 to 384 bp in length) were inserted at the anticipated Cas9 cleavage site and showed a high level of identity to sequences derived from species of the genus *Oncorhynchus* which contains various Pacific salmon species (see Fig. 2 and Additional file 1: Table S6 and Additional file 3: Figure S3). One member of this family is *Oncorhynchus keta*, the chum salmon, the source of the salmon sperm DNA used as a carrier in the yeast lithium acetate transformation procedure.

### Discussion

Microproteins present a significant fraction of the proteome in all three kingdoms of life. The 13.8 Mb genome of the genetically tractable fission yeast S. pombe potentially encodes ~ 200 proteins of 100 amino acids or fewer (~ 5% of the total proteome), twenty of which are conserved in at least one other *Schizosaccharomyces* species only and which are as yet uncharacterised. Here we report an initial investigation of the function of four of these microproteins, designated Smp1 - Smp4 for *Schizosaccharomyces* microprotein 1–4 (Table 1). Using CRISPR–Cas9 gene editing technology we first attempted to delete the four genes, *smp1*^+^–*smp4*^+^. All four gene deletion strains *smp1∆*–*smp4∆* were readily obtained, with deletion frequencies consistent with previous reports [6], indicating that all four proteins Smp1–Smp4 are non-essential for *S. pombe* haploid growth and division. Subsequent analysis of the properties of the *smp1∆*–*smp4∆* strains failed to identify sensitivity to various cellular stressors, such as temperature, osmolarity, cell wall perturbation, transcriptional inhibition or DNA damage (Additional file 1: Table S5). In addition, none of the Smp1–Smp4 proteins was essential for meiosis or sporulation (Additional file 3: Figure S2). Further work will clearly be required to provide an insight into the function of these proteins.

During PCR screening for deletion strains however, we identified a number of strains in which the PCR product obtained was noticeably larger than would be expected from wild-type cells, suggesting insertion of extraneous sequences, and several clones in which no PCR product could be obtained using standard PCR conditions, which could be due to these have inserted sequences that are too long to be efficiently amplified or to loss of primer binding sites as result of deletion of sequence on one or both sides of the targeted locus. We characterised six of the insertion strains by first determining the sequence of the inserted DNA and then database searching to determining its origins. To our surprise, all six sequenced inserts were mostly closely related to sequences derived from members of the genus *Oncorhynchus* which includes the Pacific salmon and Pacific trout. The genus also contains the chum salmon, *Oncorhynchus keta*, the source of the commercially sourced salmon sperm DNA that is routinely used as carrier DNA during yeast transformation. In each of the clones we sequenced the salmon DNA was inserted at the intended Cas9 cleavage site, most likely by the yeast non-homologous end joining (NHEJ) or microhomology-mediated end joining (MMEJ) pathways. Traditionally, salmon sperm DNA is denatured prior to use as a carrier (by heating to 100 °C and rapid cooling on ice) but we routinely omit this denaturation step and still recover more than enough transformants for downstream processing. However, our results suggest that for CRISPR/Cas9-mediated genome editing in *S. pombe*, denaturation of the carrier DNA may be highly advisable to maximise recovery of the desired deletion and reduce the background of strains with inserted salmon sperm DNA, even when the boost to transformation efficiency that results from denaturation of the carrier DNA is not crucial for subsequent steps.

## Limitations

While we have shown that the four fission yeast microproteins Smp1–Smp4 are non-essential for mitotic growth, meiosis and sporulation, and that cells lacking these proteins are not supersensitive to various cell stressors, further study is clearly required to determine their cellular function. Similarly, clarification of the molecular mechanism underlying carrier DNA insertion into the genome will require further analysis.

## Additional files

**Additional file 1: Tables S1–S6.** Yeast strains (Table S1), oligonucleotides (Table S2), plasmids (Table S3), homologous recombination templates (Table S4), phenotypic screening conditions (Table S5) and details of sequence insertions (Table S6).

**Additional file 2: Methods.** Methods for growth and phenotypic analysis of fission yeast, molecular biology and genome editing.

**Additional file 3: Figures S1–S3.** Multiple sequence alignments of Smp1–Smp4 proteins from various *Schizosaccharomyces* species (Figure S1), basic phenotypic analysis of *smp1∆*–*smp4∆* strains (Figure S2) and insertion sites relative to sgRNA and PAM sequences for all six insertion alleles (Figure S3).

## Abbreviations

SEP: smORF-encoded peptide
smORF: small open reading frame
Smp: *Schizosaccharomyces*-specific microprotein
