# Supplementary material for: Unexpected insertion of carrier DNA sequences into the fission yeast genome during CRISPR–Cas9 mediated gene deletion
Source: BMC Res Notes. 2019 Mar 29;12:191. doi: 10.1186/s13104-019-4228-x (PMC6441176; doi:10.1186/s13104-019-4228-x)
Supplement: Supplementary file 1 — Additional file 1: Tables S1–S6. Yeast strains (Table S1), oligonucleotides (Table S2), plasmids (Table S3), homologous recombination templates (Table S4), phenotypic screening conditions (Table S5) and details of sequence insertions (Table S6). [file 13104_2019_4228_MOESM1_ESM.pdf]

Additional file 1

Table S1: Yeast strains		
**No.**	**Genotype**	**Source**
Sp347	h^-	Lab collection
Sp348	h^{+S}	Lab collection
Sp938	*fex1 fex2 leu1-32 ura4-D18 ade6-M216 his3-D1* h-	[1]
Sp939	*fex1 fex2* h^{+S}	This study
Sp969	*smp1Δ fex1 fex2* h^{+S}	This study
Sp970	*smp2Δ fex1 fex2* h^{+S}	This study
Sp976	*smp3Δ fex1 fex2* h^{+S}	This study
Sp952	*smp4Δ fex1 fex2* h^{+S}	This study
Sp971	*smp1-S1 fex1 fex2* h^{+S}	This study
Sp972	*smp1-S2 fex1 fex2* h^{+S}	This study
Sp975	*smp1-S3 fex1 fex2* h^{+S}	This study
Sp979	*smp3-S1 fex1 fex2* h^{+S}	This study
Sp980	*smp3-S2 fex1 fex2* h^{+S}	This study
Sp981	*smp3-S3 fex1 fex2* h^{+S}	This study
Sp996	*smp1Δ* h^-	This study
Sp997	*smp1Δ* h^{+S}	This study
Sp998	*smp2Δ* h^-	This study
Sp999	*smp2Δ* h^{+S}	This study
Sp1000	*smp3Δ* h^-	This study
Sp1001	*smp3Δ* h^{+S}	This study
Sp1002	*smp4Δ* h^-	This study
Sp1003	*smp4Δ* h^{+S}	This study
Note: Strains Sp996-Sp1003 may also contain mutant *fex1* and/or *fex2* alleles.		

**Table S2: Oligonucleotides**	
A. PCR primers for ligation-free cloning	
SpSmp1-sgFw	5′-AAGAATAAAGGAGGGAAGACGTTTTAGAGCTAGAAATAGCAAGTTAAAATAA-3′
SpSmp1-sgRv	5′-GTCTTCCCTCCTTTATTCTTTTCTTCGGTACAGGTTATGTTTTTTGGCAACA-3′
SpSmp2-sgFw	5′-TAAGAAGAAGCAACCTATACGTTTTAGAGCTAGAAATAGCAAGTTAAAATAA-3′
SpSmp2-sgRv	5′-GTATAGGTTGCTTCTTCTTATTCTTCGGTACAGGTTATGTTTTTTGGCAACA-3′
SpSmp3-sgFw	5′-CGCACCGAAGCCGAACACGAGTTTTAGAGCTAGAAATAGCAAGTTAAAATAA-3′
SpSmp3-sgRv	5′-TCGTGTTCGGCTTCGGTGCGTTCTTCGGTACAGGTTATGTTTTTTGGCAACA-3′
SpSmp4-sgFw	5′-GAAGAAGGAGGAGTTGTATGGTTTTAGAGCTAGAAATAGCAAGTTAAAATAA-3′
SpSmp4-sgRv	5′-CATACAACTCCTCCTTCTTCTTCTTCGGTACAGGTTATGTTTTTTGGCAACA-3′
B. Primers for diagnostic PCR	
SpSmp1-CkFw	5′-TCAGGATGGATGCATCATTG-3′
SpSmp1-CkRv	5′-GGATTCACATTGGCAGCTC-3′
SpSmp2-CkFw	5′-TGTAGAAGACGATCTGTCAGCA-3′
SpSmp2-CkRv	5′-GGAGCGAGAAAACTATCGGGCT-3′
SpSmp3-CkFw	5′-CTCATTGCGGAACAGCTTTGT-3′
SpSmp3-CkRv	5′-GGCCCACTCGCTAAAGTGAA-3′
SpSmp4-CkFw	5′-ATAATGTGCCTCACCGCCAT-3′
SpSmp4-CkRv	5′-AAAGCAGACCGGAGACGAAG-3′
Notes: Underlined nucleotides in primers for ligation-free cloning correspond to sgRNA region.	

**Table S3: Plasmids**	
pJB166	[1]
pJB166-Smp1-sgRNA	This study
pJB166-Smp2-sgRNA	This study
pJB166-Smp3-sgRNA	This study
pJB166-Smp4-sgRNA	This study

**Table S4: Homologous recombination templates**
SpSmp1-gB (480 bp)
5′-AGTTCAGCAAAAACAGCTAGGGCTGTCCATAAATTCCAAAAACAACTAAAGAACACGATCGATGGATAAACCTCTCAC<br>GTTCCAAAAATTAAAGTATAACTAATTGTAGAAGAAAGAAAGCAAATAAGTAACCCAAGGAAAGGTTATTCTTCTTAAAAC<br>TTCTAAGTAACATTTTGGTAGTCAAATTGCATGATTAAAATCCGAAAAGCAGCCTTTTTTTGTATGCTGTCTTGAGAAGAA<br>ATATTTTTTTTTAAAAAAATTGCAAGGTCTCATTCGATTAATCCAGATAATCTTTTCTTTCATACACTAAAAATGGAGTTA<br>GACGTTCATATTCACCTCTCATTGAAAATGCCAAGCAAAGGCAAATGAACTGTGCTTTTTAAAAATCGAAATTCTACTTTT<br>TGATTTCACTATTAATGGATCCAGAAGAATTCGAAGGTGCTTCACGCCTTGGCGGGCCAATAAATAAGACACTAAAAG-3′
SpSmp2-gB (480 bp)
5′-TATATTAAACGTTGATGCAAAGGACACCGCTCTTCAACCATGTATTTTGTATCGAGATAAATAATTGAATATGAAATT<br>CTACCTATATGCTTAATAAAATCAAAAATTGCCTCGCATAAACAAAAAAAAACTCGGTGATTAAATACACCGAATCACTTG<br>GTTAGCCGCAAGGCAATATGAAGTGGATTGTAATGTGTTTGTTTACTAATCCATTACACCAATTTTCTTGCACACATTCAC<br>ATAAAATATAGATGCAGCTGCATCTGCAGCATATTGATGGCGTGCTTGCACATACTCCTCAACCCTTCAAACTTTTACTTC<br>AATAAACGAAGGGTACCAAACCACTACCTTTCTTGAACTATTTTATCAAAGAAATGCATAATAGCATTAACGAATAATGAA<br>AATTACTACCAAACCATTTTGCTTGCCAACAACAATTGAGTTTGTGTAACTTGATATATACTAGAAGATAAATCTAAA-3′
SpSmp3-gB (480 bp)
5′-TCATAAATCCAATTCCTTTAATTATTCATAAGACCAATTTTTGGTTAATTTTCTTGTTCGCTAAGAACTACCTTAATT<br>GTGGACCCCGTTGAATCAACCTTCTTTTTATATTGATCAACGAACGAGCAATTCGATTAGGACTAGCATATCCTTGGTTTT<br>CTTGCGTGTTCAATTACTAAAAACCCTGTTTTCTGTTTGTTAATATATATTTTTTTACTCTCCCTCTCTTAGGTATACAGG<br>AAAGGGAGGTGGGAATGGAGCGAGGAAGCCATTCTTCTTTATAAATAATTTGCTCCCTTTGTCCTCCTTATTAGTTTATTG<br>GAGGACGTTTACTAGATGATTACTTTTTAATTACGAAACGACTTATTTATATAATCGGTACATGTTTTCCTTCCTGTTCGC<br>ATTCATGCATGTTCCTCGTGAAGTTCATTTTATTATGATGATTATTGCTCTCACGTTTGTCTTTTATTGATTTCCTTA-3′
SpSmp4-gB (500 bp)
5′-TGAGTTGTACATCATATGTTGACGTCATTTACGGTATGTTGAAATAGTATATAGCAGCAAGTGAAACCGCAGAAGTTG<br>ATTTCATTCTCCATACATTCATTCTCAAATACGAATTGTAATTGTAAAAAGACAATTAAATATTCGCATTTTTACAAGATT<br>CAGCATTTCCTATATTAATTTGGAATAAATCTTTTTTTGACCACATTTTTTTCCAGTTGTCATACTCGTTTCTCTAAATAA<br>ACTATCAATAAAGAAGTGCATTCAATTAACAAACTAAGTATAATCAACAATTACTAAAATTCTACGCTTAATTCGTGATGC<br>CTTTTTTTGATCGATGATTTCATGCCTCATGACTTTTAGATACCAAAAAAAATGTTAATGACTAATGAAAGAACACCTTAA<br>ATCTTATATGAAAATGAATTTTTGCCCAAACGTAATATTAAAACTATACTAAGTGGATGTCGCACACGTATGTGTATTTAA<br>TAGTATCGCCAGTGTAA-3′
Note: 5’ flanking sequences in red, 3’ flanking sequences in blue.

Table S5: Phenotypic screening conditions	
**Stressor**	**Conditions tested**
Temperature	20°C, 25°C, 28°C, 32°C, 35°C
KCl	0.25 M, 0.5 M, 1 M
Sorbitol	0.3 M, 0.6 M, 1.2 M
SDS	0.0025%, 0.005%, 0.01%, 0.02%
6-azauracil	0.2 mM, 0.4 mM, 0.8 mM
Calcofluor White	0.25 mg/ml, 0.5 mg/ml, 1 mg/ml
TBZ	6.25 μg/ml, 12.5 μg/ml, 18 μg/ml, 25 μg/ml
Hydroxyurea	0, 3, 6, 9 and 12 mM
MMS	0.005%, 0.0065%, 0.008%, 0.01%
EGTA	2.5 mM, 5 mM, 10 mM, 15 mM
UV	0, 100, 200, 300, 400 J/m^2

Table S6: Insertions of *Oncorhynchus* sequences at targeted loci		
Insertion allele	Insertion size	Closest match in sequence databases
smp1-S1	384 bp	*Oncorhynchus tshawytscha*, 92% identity over 399 bp, GenBank: PIPH01205116.1
smp1-S2	183 bp	*Oncorhynchus tshawytscha*, 96% identity over 185 bp, GenBank: PIPH01065853.1
smp1-S3	73 bp	*Oncorhynchus tshawytscha*, 97% identity over 70 bp, GenBank: PEKY01000129.1
smp3-S1	121 bp	*Oncorhynchus kisutch*, 84% identity over 122 bp, GenBank: MPKV01002807.1
smp3-S2	121 bp	*Oncorhynchus tshawytscha*, 98% identity over 120 bp, GenBank: GenBank: MPKV01003120.1
smp3-S3	113 bp	*Oncorhynchus mykiss*, 98% identity over 113 bp, GenBank: GenBank: MSJN01000047.1
BLAST searching was performed using blastn default parameters at blast.ncbi.nlm.nih.gov and the WGS (whole genome shotgun) sequence database.		

References

1. Fernandez R, Berro J: Use of a fluoride channel as a new selection marker for fission yeast plasmids and application to fast genome editing with CRISPR/Cas9. *Yeast* 2016, 33:549-557.
