# Supplementary material for: Unexpected insertion of carrier DNA sequences into the fission yeast genome during CRISPR–Cas9 mediated gene deletion
Source: BMC Res Notes. 2019 Mar 29;12:191. doi: 10.1186/s13104-019-4228-x (PMC6441176; doi:10.1186/s13104-019-4228-x)
Supplement: Supplementary file 2 — Additional file 2: Methods. Methods for growth and phenotypic analysis of fission yeast, molecular biology and genome editing. [file 13104_2019_4228_MOESM2_ESM.pdf]

Additional file 2

Methods

Fission yeast strains and growth medium

The *S. pombe* strains used in this study are listed in Additional file 1, Table S1. Strain Sp939 was constructed by crossing *fex1*$^-$ *fex2*$^-$ *leu1-32 ura4-D18 ade6-M216 his3-D1* h$^-$ [1] with Sp348 (wild-type h^{+S}) and identifying fluoride-sensitive (*fex1*$^-$ *fex2*$^-$) prototrophs from among the progeny, with the mating type being determined by PCR [2]. *S. pombe* was routinely grown in YE4S medium or on YE4S agar plates (supplemented as required) at 32°C [3]. Matings were performed using SPA agar at 25°C [4].

Molecular biology reagents and methods

PCR for ligation-free cloning was performed using either Q5 or Phusion DNA polymerase (New England Biolabs) in a ^{3}Prime Thermal Cycler (Techne). Chemically-competent NEB® 10-beta cells were purchased from New England Biolabs. Plasmid DNA was prepared using the QIAprep Spin Miniprep Kit (Qiagen). Genomic DNA for diagnostic PCR was prepared from single colonies as described [5]. Genomic DNA for PCR and subsequent sequencing was prepared from ~ 1 x 10^7 exponentially growing cells using the YeaStar™ Genomic DNA kit (Zymo Research). Diagnostic PCR was performed using MyTaq™ Red Mix (Bioline) in a Piko Thermal Cycler (ThermoFisher). DNA sequencing was performed by DNA Sequencing & Services (University of Dundee) using Applied Biosystems Big-Dye Ver 3.1 chemistry on an Applied Biosystems model 3730 automated capillary DNA sequencer.

Plasmid construction

Plasmids encoding sgRNAs were constructed by ligation-free cloning as described [6]. Briefly, 5 ng of plasmid pJB166 [1] was amplified in a 50 µl PCR reaction using either Q5 or Phusion high-fidelity DNA polymerases and 1 µM forward and reverse primers designed by CRISPR4P [6]. Primer sequences are listed in Additional file 1, Table S2. Cycle parameters for Q5 DNA polymerase reactions: 98°C for 30 seconds (initial denaturation), then 25 cycles of 98°C for 10 seconds, 60°C for 20 seconds and 72°C for 7 minutes, followed by 72°C for 7 minutes (final extension). Cycle parameters for Phusion DNA polymerase reactions: 98°C for 2 minutes (initial denaturation), then 25 cycles of 98°C for 10 seconds, 60°C for 30 seconds

and 72°C for 5 minutes 30 seconds, followed by 72°C for 5 minutes (final extension). 5 μl of the PCR reaction was then transformed directly into chemically-competent NEB® 10-beta cells (New England Biolabs), plated on LB agar plates supplemented with 100 μg/ml ampicillin and incubated overnight at 37°C. The use of NEB® 10-beta appeared to prevent the previously described instability of plasmid pJB166 thereby eliminating the need for pre-screening of bacterial clones prior to plasmid preparation [6]. Colonies were then transferred to 5 ml of LB medium supplemented with 100 μg/ml ampicillin and grown for 16 hours at 37°C with shaking. Miniprep DNA was prepared and analysed by restriction with HindIII to confirm the absence of major rearrangements of plasmid DNA. The sgRNA-encoding insert was then sequenced using standard M13 forward and reverse primers.

Transformation and PCR screening

Preparation of competent cells, cryopreservation and transformation of *S. pombe* was as described previously [6]. For transformation, 50 μl aliquots of cryopreserved G1-arrested cells were transformed with 2 μg of plasmid DNA (10 μl of a 200 ng/ml solution), 100 ng of HR template DNA (10 μl of a 10 ng/μl solution, purchased as a gBlock® Gene Fragment, Integrated DNA Technologies), and 20 μg of UltraPure™ Salmon Sperm DNA (2 μl of a 10 mg/ml solution, Invitrogen). Following overnight incubation in EMM-N medium at room temperature without shaking, the cells were washed once in EMM-N and plated onto two YE4S agar plates supplemented with 1 mM sodium fluoride. Plates were incubated for 4 days at 32°C, at which point 24-32 of the smallest transformant colonies were picked and re-streaked onto YE4S medium to allow loss of the pJB166-derived sgRNA plasmid. After 3 days, at least 16 single colonies were picked and chromosomal DNA prepared and used as a template in diagnostic PCR reactions [5] using MyTaq™ Red Mix (Bioline). Primers for screening for deletions were designed by the CRISPR4P tool (see Additional file 1, Table S2 for primer sequences). Colonies of interest (i.e. where the size of the PCR product was different from expected wild-type size) were then re-streaked on YE4S and re-tested by diagnostic PCR with wild-type controls. Finally, genomic DNA was prepared from exponentially growing cells using the YeaStar™ Genomic DNA Kit (Zymo Research), amplified using MyTaq™ Red Mix (Bioline) and the PCR products was sequenced using primers located outside the HR template region (see Additional file 1, Table S2). Strains were subsequently stored at -80°C in 20% glycerol.

Phenotypic analysis

Cells were grown to mid-exponential phase overnight at 32°C with shaking in YE4S medium. Growth rates at 32°C in YE4S were determined by monitoring OD_{600nm} over 8 hours using a Biowave CO 8000 Cell Density Meter (Biochrom WPA), starting from 0.1 (approximately 1 x 10^6 cells/ml). Spotting assays were performed by preparing 5X serial dilutions in YE4S medium and spotting 3.0 µl aliquots onto YE4S plates supplemented as necessary with various cell stressors (see Table S5 for concentration ranges). For UV sensitivity assays, spotted plates were irradiated with UV light (254 nm) using a Stratalinker® 2400 UV crosslinker (Stratagene).

Meiosis and sporulation

Cells were plated on EMM agar and incubated at 25°C overnight, before being resuspended in sterile H_2O, mixed and spotted on SPA agar [4]. Asci were visualised after 48 hours at 25°C.

References

1. Fernandez R, Berro J: Use of a fluoride channel as a new selection marker for fission yeast plasmids and application to fast genome editing with CRISPR/Cas9. *Yeast* 2016, 33:549-557.
2. Ekwall K, Thon G: Mating-type determination in *Schizosaccharomyces pombe*. *Cold Spring Harb Protoc* 2017, 2017:pdb prot091728.
3. Moreno S, Klar A, Nurse P: Molecular genetic analysis of fission yeast *Schizosaccharomyces pombe*. *Methods Enzymol* 1991, 194:795-823.
4. Ekwall K, Thon G: Setting up *Schizosaccharomyces pombe* crosses/matings. *Cold Spring Harb Protoc* 2017, 2017:pdb prot091694.
5. Looke M, Kristjuhan K, Kristjuhan A: Extraction of genomic DNA from yeasts for PCR-based applications. *Biotechniques* 2011, 50:325-328.
6. Rodriguez-Lopez M, Cotobal C, Fernandez-Sanchez O, Borbaran Bravo N, Oktriani R, Abendroth H, Uka D, Hoti M, Wang J, Zaratiegui M *et al*: A CRISPR/Cas9-based method and primer design tool for seamless genome editing in fission yeast. *Wellcome Open Res* 2016, 1:19.
