# Supplementary material for: Unexpected insertion of carrier DNA sequences into the fission yeast genome during CRISPR–Cas9 mediated gene deletion
Source: BMC Res Notes. 2019 Mar 29;12:191. doi: 10.1186/s13104-019-4228-x (PMC6441176; doi:10.1186/s13104-019-4228-x)

Additional file 3

Figure S1: Multiple sequence alignment of Smp1-Smp4 proteins from different *Schizosaccharomyces* species. Abbreviations: Sp, *S. pombe*; So, *S. octosporus*; Sj, *S. japonicus*; Sc, *S. cryophilus*. Homologues were identified by BLAST searching the UNIPROTKB_FUNGI database at the Universal Protein Resource (UniProt). Alignments were generated using Clustal Ω (1.2.4).

```
SpSmp1     MSPRASLEKELNSARLLHATINAMDVYTQNLINELQEARDSINDLQRAHERLKLVGAKAKLQIKRDEKKPKS   72
SjSmp1       MQQHRKQQLKEAKMLHATVNAVDLYLSELLEDIRASSDAIKKLDTSENKFENTLSQA---IKKTGKNA--   65
                : :::*:.*::****:**:*:* .:*::::: : *:*:.*: :.:::: . ::*   **:  *:

SpSmp2               MQHNKENHFVEDAKQFQEKAKLYQGNYYTLDGELITIIPSSKEGFRSCKSLYYKKKQPIPGR   62
ScSmp2               MEQDKENQLNLPKLSIPQDIKVFQGNYYTLDGEQTP-NPDGTTEFKTCKALYYKSKKPIPGR   61
                     *:::***::     .: :. *::**********     *...  *::**:****.*:*****

SpSmp3                         MAQTFQEKQQSRRIKMSTGNFFSRMWNAVVFGFGAAIGASVANAALGACCG-   51
SjSmp3                          MATFQEKQGQRRIKMRTGNFFSRMWNAVIFGFGAAIGATVANAALGACCG-   50
ScSmp3                         MGRTFQEKQQSRRVKMSTGNIFSRVWNSVVFGFGAALGATAASAMIGACCG-   51
SoSmp3                         MARTFQEKQQGRRMKMSTGNIFSRVWNSVVFGFGAALGATAASAMLGACCGG   52
                                  ****** **:** ***:***:**:*:******:**:.*.* :*****

SpSmp4        MSAPYKNLDRDTKHTHPKLNETERNLNRGWGDVKKEELYEDLAQSDADKQLAEDKMETKYEKSKPAPSD   69
SoSmp4       MSPAPYKDLSKDTVHTHPPIHESERREEHGWGSLKKEPLMEDQAEQDAKADLSKDKKEVKEEASRPPPSV   70
ScSmp4       MSPAPYKDLSKDTVHTHPAIRESERREQHGWGDLNKEPLMEDQAERDAKNDLSKDQKEVKEQASRPAPNN   70
SjSmp4    MPYMPQVKHGEVIRDDRHSHTGLKISDRADTRGWGDVKHEREFEELAERDAETDQRRDTEEVQKEPELLPPQ-   72
                . : :: :*  *:*  :. ::*   :***.:::*   *: *: **. :  .*  *.: : .   *.
```

Figure S2: Phenotypic analysis of *smp1*Δ - *smp4*Δ cells. **A.** Microscopic images of wild-type, *smp1*Δ, *smp2*Δ, *smp3*Δ and *smp4*Δ cells growing exponentially in YE4S medium at 32°C. Scale bar: 10 µm. **B.** Growth rates (OD_{600nm} versus time) of wild-type (WT), *smp1*Δ, *smp2*Δ, *smp3*Δ and *smp4*Δ cells growing in YE4S medium at 32°C. **C.** Examples of meiotic asci formed by self-crossing wild-type, *smp1*Δ, *smp2*Δ, *smp3*Δ and *smp4*Δ strains of opposite mating type on SPA agar at 25°C for 48 hours. The figure was assembled from multiple representative images of the same crosses. Scale bar: 10 µm.

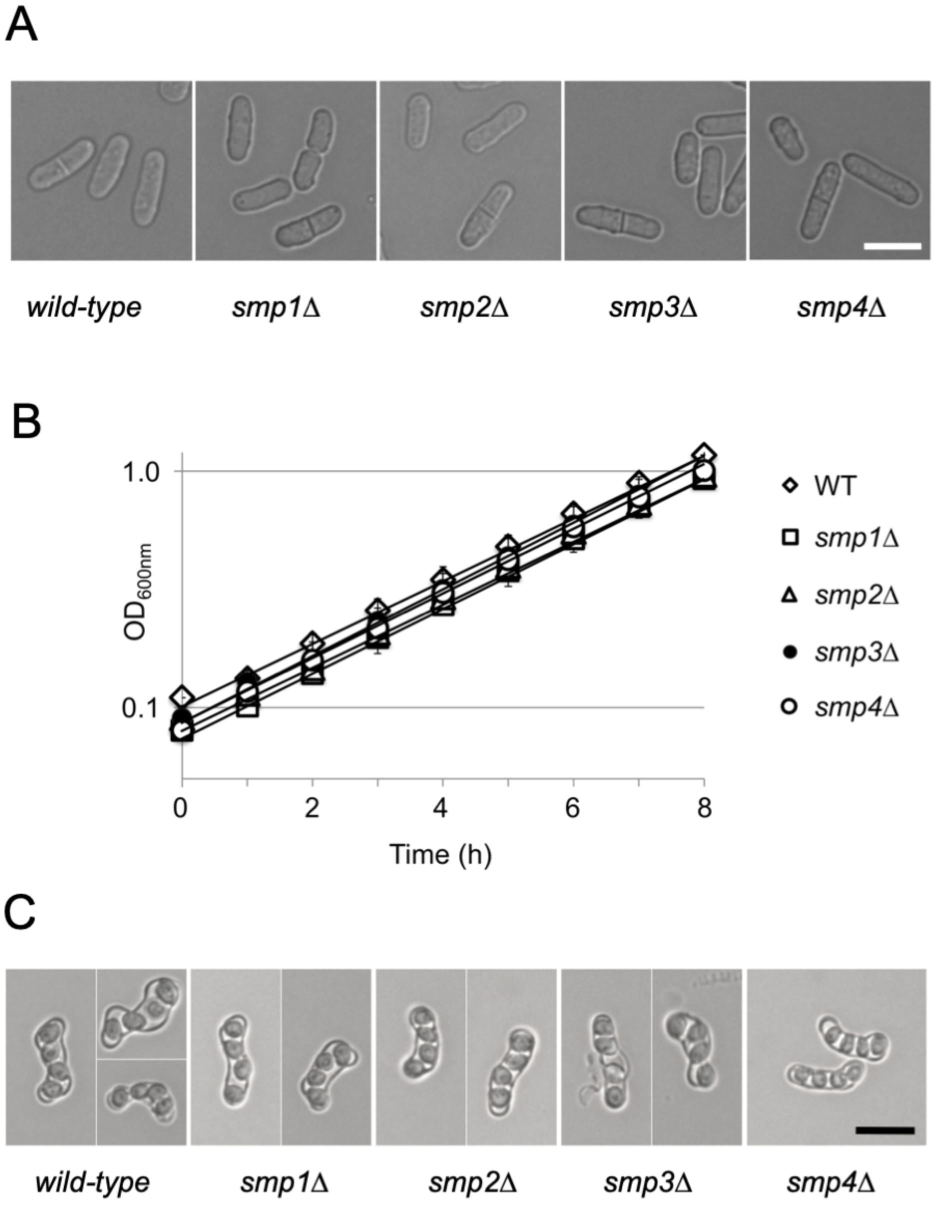

Figure S3: Insertion sites relative to sgRNA and PAM sequences. Sequences of *smp1-S1 – smp1-S3* and *smp1-S1 – smp3-S3* alleles are shown. PAM sequences are shown in bold, sequence corresponding to sgRNA in red. Note that the insertion in *smp-S3* also deletes two nucleotides. See Table S6 for identity of inserted *Oncorhynchus* sequences.

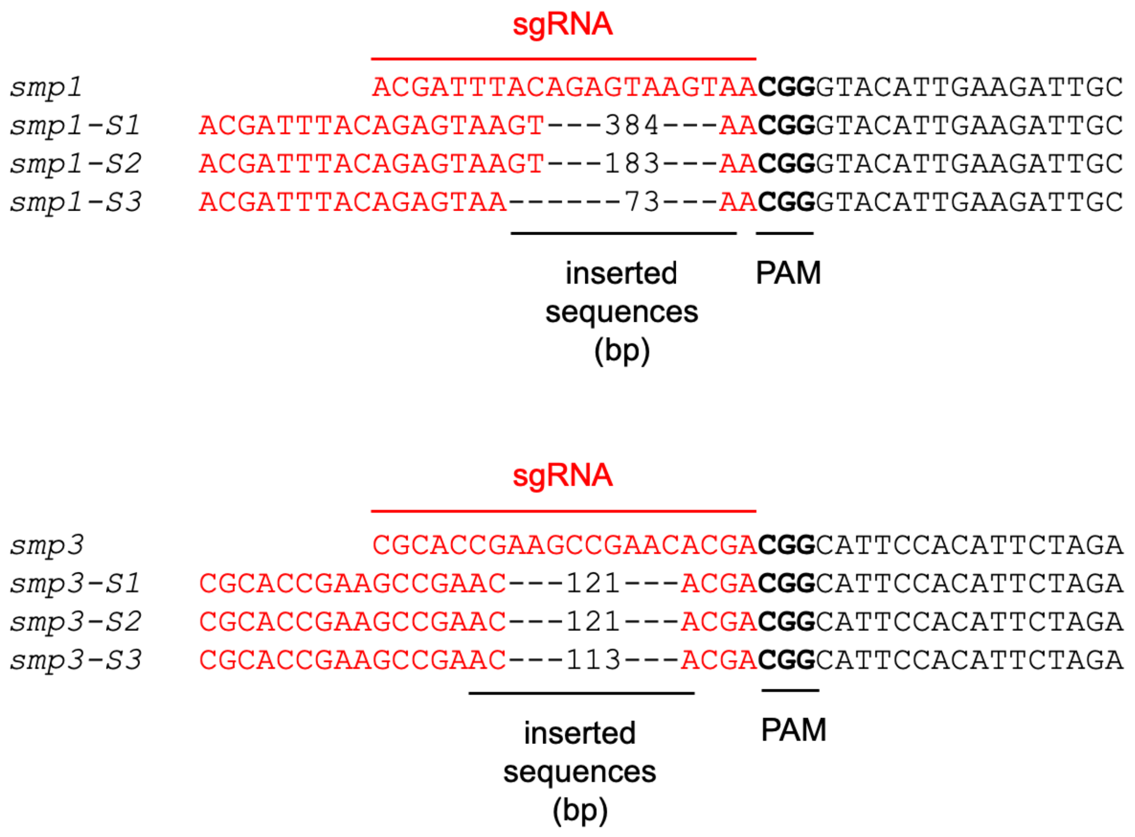

Supplement: Supplementary file 3 — Additional file 3: Figures S1–S3. Multiple sequence alignments of Smp1–Smp4 proteins from various Schizosaccharomyces species (Figure S1), basic phenotypic analysis of smp1∆–smp4∆ strains (Figure S2) and insertion sites relative to sgRNA and PAM sequences for all six insertion alleles (Figure S3). [file 13104_2019_4228_MOESM3_ESM.pdf]
